# Targeting PI3K/Akt/mTOR Pathway, Ki-67 and Endothelin Receptors by Ambrisentan in Juvenile Rat Intestinal Ischemia

**DOI:** 10.3390/ijms27125370

**Published:** 2026-06-14

**Authors:** Marwa Monier Mahmoud Refaie, Hanaa Hassanein Mohammed, Asmaa A. Hasan, Sayed Shehata, Asmaa A. Muhammed, Mohamad Assayed Nader, Zamzam M. Abdelsamie, Hoda S. Sherkawy, Enas Fathy, Shereen Mohammed Mohammed Elsaghir, Fatma F. Ali, Ahmed M. Ashour, Ali Khames, Doaa Mohamed Elroby Ali

**Affiliations:** 1 Department of Medical Pharmacology, Faculty of Medicine, Minia University, Minia 61511, Egypt; marwamonier@mu.edu.eg; 2 Faculty of Medicine, Minia National University, New Minia 61768, Egypt; hanaa.hasanian@minia.edu.eg; 3 Department of Histology and Cell Biology, Faculty of Medicine, Minia University, Minia 61511, Egypt; 4 Department of Human Anatomy and Embryology, Faculty of Medicine, Aswan University, Aswan 81528, Egypt; asmaa.elsaid@aswu.edu.eg; 5 Department of Cardiology, Faculty of Medicine, Minia University, Minia 61511, Egypt; sayedshehata1@mu.edu.eg; 6 Department of Medical Physiology, Faculty of Medicine, Aswan University, Aswan 81528, Egypt; asmaa.ali@med.aswu.edu.eg; 7 Department of Physiology, RAK College of Medical Sciences, RAK Medical & Health Sciences University, Ras-AlKhaimah 11127, United Arab Emirates; 8 Pediatric Surgery Unit, General Surgery Department, Faculty of Medicine, Aswan University, Aswan 81528, Egypt; mnader1st@aswu.edu.eg; 9 Department of Pediatric, Faculty of Medicine, Aswan University, Aswan 81528, Egypt; zamzam.mansour@aswu.edu.eg; 10 Department of Medical Biochemistry, Faculty of Medicine, Aswan University, Aswan 81528, Egypt; hoda.sultan42@aswu.edu.eg; 11 Department of Cardiology, Faculty of Medicine, Aswan University, Aswan 81528, Egypt; enas.fathy@aswu.edu.eg; 12 Department of Internal Medicine, Faculty of Medicine, Minia University, Minia 61511, Egypt; sherine.al-saghir@mu.edu.eg; 13 Department of Biochemistry, Molecular biology and Physiology, Faculty of Medicine, Mutah University, Al-Karak 61101, Jordan; fatma.ahmed2@mu.edu.eg; 14 Department of Medical Physiology, Faculty of Medicine, Minia University, Minia 61511, Egypt; 15 Department of Pharmacology and Toxicology, College of Pharmacy, Umm Al-Qura University, Makkah 21955, Saudi Arabia; amashour@uqu.edu.sa; 16 Department of Pharmacology and Toxicology, Faculty of Pharmacy, Sohag University, Sohag 82524, Egypt; 17 Department of Biochemistry and Molecular Biology, Faculty of Pharmacy, Sohag University, Sohag 82524, Egypt; doaa.elroby@pharm.sohag.edu.eg; 18 Department of Biochemistry, Faculty of Pharmacy, Badr University Branch Assiut, Assiut 71511, Egypt

**Keywords:** ambrisentan, intestinal ischemia–reperfusion, endothelin, protein kinase B

## Abstract

Juvenile intestinal ischemia–reperfusion (JII/R) is a pediatric surgical emergency caused by mesenteric vessel occlusion and has a high mortality rate. Malrotation can cause intestinal ischemia in infants due to midgut volvulus. It affects not only the intestine itself but also other organs, such as cardiac tissue. Therefore, searching for more effective therapeutic solutions is an essential critical need. This directed our thoughts to evaluate the role of ambrisentan (AMB) in a rat model of induced JII/R by clamping the superior mesenteric artery. Forty juvenile male Wistar albino rats (3–4 weeks old) were randomly divided into four experimental groups: control (CON) group, JII/R group, and AMB-treated groups (30, 60 mg/kg) with JII/R. Induction of JII/R results in significant changes in cardiac enzymes, oxidative stress, inflammatory, and apoptotic parameters with high expression of endothelin receptor A (ERA). Also, histopathological changes revealed extensive mucosal damage, loss of intestinal villi, dysregulated and degenerated cardiac fibers with inflammatory cell infiltration, and tissue necrosis. In contrast, AMB administration significantly reduced the elevated levels of cardiac enzymes, malondialdehyde (MDA), nuclear factor kappa B (NF-κB), ERA, and caspase-3 expression. However, AMB treatment increased immune expressions of phosphatidylinositol 3-kinase (PI3K), protein kinase B (Akt), Ki-67, and mammalian target of rapamycin (mTOR) and showed remarkable improvement in the histopathological changes. AMB could be considered as an adjuvant medical treatment for cases of JII/R.

## 1. Introduction

The intestinal tract is a major system responsible for the safe absorption of different necessary nutrients for life. It is exposed to harmful pathogens, and its ability to constrain them is dependent on sufficient oxygenation and blood supplies [1]. The intestinal mucosal barrier comprises a series of chemical, biological, mechanical, and immune defense mechanisms standing against the translocation of these harmful substances and pathogens into the systemic circulation [1]. Therefore, intestinal damage, as in cases of intestinal ischemia, leads to bacterial translocation, followed by sepsis and multiple organ failure [2,3,4].

Intestinal ischemia is a common clinical disorder characterized by a marked reduction in mitochondrial oxygen uptake, falling below the threshold level necessary to maintain the normal health of intestinal cells and ensure proper oxidative metabolism [2,3]. It is not limited to the intestinal tissue itself, but it can also lead to further systemic inflammatory responses and multiple vital organ dysfunctions with high mortality rates. Researchers have studied the significant impact of intestinal ischemia on the cardiovascular system, even after surgical intervention [4,5]. Bowel ischemia and cardiac injury are commonly linked together due to the shared risk factors [3,4,5].

Intestinal ischemia–reperfusion (II/R) is a peculiar emergency case in some neonates and newborns. This condition develops as the inflammatory processes are activated, and the compensatory saving mechanisms are still immature, which may result from different clinical conditions, including neonatal necrotizing enterocolitis (which accounts for about 5–7% of II/R cases in neonates), intestinal obstruction, midgut volvulus, strangulated hernia and shock, and incarcerated hernia [6,7,8,9,10].

Several theories elucidate that juvenile intestinal ischemia reperfusion (JII/R) injury triggers an oxidative stress response characterized by the excessive release of harmful agents leading to tissue damage [6,7,8,9,10]. Nevertheless, the restoration of blood supply during surgical manipulation and tissue reperfusion is accompanied by destructive molecular events, including the release of reactive oxygen species (ROS), tumor necrosis factor α, interleukin 1β, and nuclear factor kappa b (NF-κB), along with the infiltration of polymorphonuclear neutrophils that disrupt epithelial cell homeostasis [11,12]. In addition, the phosphatidylinositol 3-kinase/protein kinase B/mammalian target rapamycin (PI3K/AKT/mTOR) cascade and Ki-67 have great importance in regulating different cellular processes, including cell transcription, apoptosis, migration, proliferation, mitochondrial permeability transition pores, and protein synthesis [13,14].

The serine–threonine protein kinase Akt is a key downstream regulator of PI3K, triggering a cascade of essential intracellular signaling events [15]. Accordingly, the PI3K/Akt pathway plays an important role in protecting intestinal tissues during JII/R by promoting vasodilation and angiogenesis, enhancing endothelial nitric oxide synthase (eNOS) with the production of nitric oxide (NO), and preventing oxidative stress and apoptosis [16,17]. Furthermore, one of the most potent vasoconstrictor agents is endothelin (ET), which mediates its biological and pharmacological effects via activating endothelin receptor A (ERA), significantly contributing to ischemia–reperfusion pathophysiology through the enhancement of vasoconstriction and several inflammatory and apoptotic pathways [18,19]. Regulation of the PI3K/AKT/mTOR cascade and Ki-67 have been approved to reduce myocardial I/R injury, and there is a significant link between the modulation of ET receptors and the activation of the PI3K/AKT/mTOR pathway and Ki-67 [14,15,16,17,18,19,20,21]. Thus, inhibition of ET receptors and controlling such a cascade could prevent JII/R damage.

Ambrisentan (AMB) is a selective ERA antagonist used mainly to control pulmonary hypertension, and it improves hepatic, pancreatic, cerebral, cardiac, and renal microcirculation during ischemia–reperfusion and renal dysfunction [22,23,24]. AMB is well approved to have a potent suppressing effect on endothelin receptor A expression and activity, which plays a pivotal role in mediating various ischemic injuries, as ERA is highly expressed during ischemia and enhances different inflammatory pathways, oxidative stress, and apoptosis [22,23,24]. AMB is now approved by the European Medicines Agency to be safely used in children (aged 8–17 years) with pulmonary hypertension at dosages of 5–10 mg once daily based on body weight. To date, the pharmacokinetic profile of AMB has been comparable between pediatric and adult populations. No specific hazards of AMB in children have been documented [23,25,26,27]. Based on these data, we found that AMB could be beneficial and safe for ameliorating JII/R, considering the strong link between the release of ET and inflammatory mediators, as well as the presence of apoptosis, oxidative damage, and endothelial dysfunction during JII/R pathogenesis. Meanwhile, AMB has potent ERA antagonist properties and suppresses inflammation, oxidative stress, and apoptosis. This guided us to evaluate for the first time the novel medical role of AMB in JII/R-induced alimentary and cardiac injuries.

## 2. Results

### 2.1. Evaluation of the Detected Cardiac Enzymes in JII/R Model

The untreated JII/R group showed significantly higher cardiac enzyme levels than the CON non-ischemic group, while with the administration of AMB in two different doses, their serum levels diminished significantly compared to the untreated JII/R group (Table 1).

### 2.2. GSH, MDA Measurement, and TAC Evaluation

The untreated JII/R group demonstrated elevated MDA levels and decreased GSH and TAC levels in comparison to the CON group. AMB administration mitigated this effect in a dose-dependent manner (Table 2).

### 2.3. ERA and Phosphorylated mTOR Measurement in Tissues

Untreated ischemic rats in the JII/R group demonstrated significantly elevated ERA expression but decreased mTOR in comparison to CON rats. AMB (30 and 60 mg/kg) given to rats reduced the highly expressed ERA during JII/R but increased mTOR compared to the untreated JII/R animals in a dose-dependent manner (Table 3).

### 2.4. Phosphorylated PI3K and Akt in Intestinal and Cardiac Tissues

II/R significantly decreased PI3K and Akt in intestinal and cardiac tissues relative to CON rats. Nevertheless, AMB treatment increased them compared to the JII/R untreated group (Table 4).

### 2.5. Light Microscopic Results

#### 2.5.1. Hematoxylin and Eosin (H&E) Results

Regarding histological examination, the jejunal CON group showed normal villi with no inflammatory cell infiltration in the epithelial layer (Figure 1a). The JII/R group showed extensive mucosal damage, loss of villi, disintegration of the lamina propria, and hemorrhage with inflammatory cell infiltration. Local necrosis of epithelium, intestinal apical denudation of the villi, epithelial separation from the basement membrane at the base of the villi, and damage of the connective tissue were detected (Figure 1(b1–b3)). Treatment with a low dose of AMB improved most of these alterations, showing only some inflammatory cell infiltration (Figure 1c), while the high dose of AMB showed more normal jejunal histology (Figure 1d).

Left ventricles in the CON group showed normal cardiac muscle cells regularly arranged, branched, and anastomosed, forming a three-dimensional meshwork. Their sarcoplasm was acidophilic and striated with a single central oval and vesicular nucleus. Narrow spaces of connective tissue endomysium were seen between cardiac muscle cells. Few fibroblasts with flat, dense nuclei were noticed at the periphery of myocytes (Figure 2a).

In the JII/R group, cardiac muscle fibers were irregularly arranged, with areas of fiber loss, inflammatory cell infiltration, wavy fibers, hypereosinophilic fibers with deeply stained pyknotic nuclei, fragmented pale muscle fibers, and vascular congestion (Figure 2(b1–b3)). The low dose of AMB improved most of these changes, showing fewer inflammatory cells, wide areas of preserved cardiac fibers, central oval vesicular nuclei, and few areas of damaged fibers (Figure 2c). The high dose of AMB showed remarkably improved histological features (Figure 2d).

#### 2.5.2. Immunohistochemical Results in Jejunal and Cardiac Tissue

PI3K, Akt, and Ki-67 immune expressions were positive in the CON group, reflecting cell survival and normal proliferation. Faint immune expression with tissue damage was seen in the JII/R group, indicating tissue degeneration and cell death. The low-dose AMP-treated rats showed some restoration of positive immune expression in the cytoplasm, while the animals treated with high-dose AMB showed strong positive immune expressions, reflecting cell regeneration with improvement in cell proliferation and survival (Figure 3, Figure 4 and Figure 5).

#### 2.5.3. Semi-Quantitative Examination of the Histopathological and Immunohistochemical Results 

There was significant diminution of PI3K, Akt, and Ki-67 immune expressions but an increase in the histopathological score in the JII/R group compared to the CON group. However, AMB-treated groups with low or high doses showed significantly improved histopathological scores and returned the normal balance of PI3K, Akt, and Ki-67 immune expressions relative to JII/R untreated animals in both cardiac and jejunal tissues compared to JII/R untreated rats (Figure 6 and Figure 7).

### 2.6. Western Blotting Expression of Cleaved Caspase-3 and NF-κB p65

Phosphorylated NF-κB and cleaved Caspase-3 were significantly elevated in the untreated ischemic rats compared to control ones. However, both parameters significantly decreased in AMB-administered animals (30 and 60 mg/kg) compared to the JII/R untreated group (Figure 8 and Figure 9).

## 3. Discussion

Intestinal ischemia is one of the most critical clinical cases, especially in neonates and during childhood. Surgical intervention is mandatory, but hazards of ischemia are not adequately managed even after the return of blood supply [1,22,23,28]. Young children and neonates suffer from this clinical disorder due to variable situations: necrotizing enterocolitis, incarcerated hernia, and intestinal obstruction [6,7]. Thus, there is an increasing need to discover an adjuvant medical treatment acting alongside the surgical one to alleviate the hazards of intestinal ischemia either in the alimentary tract or in distant vital organs, especially in cardiac tissue. This led us to consider whether AMB would ameliorate intestinal ischemia–reperfusion injury in juvenile rats. Our findings showed significant elevations in the heart function enzymes (CK-MB, LDH, and troponin), MDA, Caspase-3, ERA and NF-κB. However, there are significant decreases in PI3K, Akt, mTOR, Ki-67, TAC, and GSH with obvious pathological changes in the histopathological examination. However, AMB administration could control ERA receptors, increase PI3K, Akt, and Ki-67 immune expressions, elevate mTOR levels as measured by ELISA, and decrease cardiac enzymes with the suppression of apoptosis, oxidative stress, and inflammation.

The JII/R-induced tissue injury is not sufficiently understood, but the tremendous release of ROS has an essential impact and stimulates various inflammatory mediators, leading to the initiation of apoptosis and cell death [7,26,27,28,29,30]. Moreover, inadequate blood supply causes tissue hypoxia with disturbance of the PI3K/Akt/mTOR pathway and the Ki-67 cascade [14,15,16,17,18,31,32,33]. Assessing oxidative stress is based on measuring MDA levels for detecting the emergence of all free radicals. On the other hand, the primary intracellular guarding mechanism against these radicals is activated antioxidants, including vital molecules such as GSH, which plays an important role in preventing oxidative damage. The JII/R group had high MDA levels but low GSH and TAC levels because of the already released free radicals [34,35,36]. In addition, apoptosis plays a significant role during JII/R injury, and the damaged mitochondria release pro-apoptotic factors and activate Caspase-9 and Caspase-3 [1,2,3,4,5]. This supports our data that found significant elevation of NF-κB, the stimulating factor of both inflammatory processes and apoptosis, with elevation of the active form of Caspase-3, the main indicator of apoptosis. Besides that, specific heart enzymes are very important in evaluating cardiac function. During JII/R, oxidative stress leads to membrane lipid peroxidation and further cell damage accompanied by the release of intracellular enzymes and elevation of their serum levels [33,34,35,36]. These findings were confirmed in the current research, which showed a significant increase in cardiac-specific enzymes in the JII/R group.

Among the key regulators in controlling ischemic injury are the PI3K/Akt/mTOR pathway and Ki-67, which play an important role in modulating various basic cellular activities, including cell growth, metabolism, proliferation, angiogenesis, and apoptosis. PI3K is activated by extracellular ligands and then catalyzes PIP2 phosphorylation in position 3 of the inositol ring, forming PIP3, which recruits AKT and PDK1 to the plasma membrane. Once AKT is gathered on the cell membrane, it is phosphorylated by mTOR complex 2, and the activated AKT loses its connection to the cell membrane and phosphorylates other essential target proteins. In addition, it has been found that the PI3K/Akt/mTOR/Ki-67 cascade is an important functional regulator of TLR4/NF-κB activity in the inflammatory signaling pathway, and it also plays a key role in ameliorating apoptosis, inflammation, oxidative stress, immune modulation, and cell survival. The results of our study showed that the immune expressions of PI3K, Akt, Ki-67, and mTOR levels by ELISA significantly diminished in JII/R, reflecting abnormal cell proliferation, degeneration, and death, and this is in accordance with previous studies [37,38,39].

In more detail, initiation of this pathway inhibits apoptotic cell death in different ways, such as diminishing death gene expression with regulation of Bcl-2 [39,40]. Recently, several studies have verified that activation of the PI3K/Akt signaling pathway has a vital role in the protection against epithelial cell apoptosis and mucosal damage, and regulating the adaptive immune response [41,42]. For example, many PI3K heterodimers critically govern cell survival, proliferation, B- and T-cell signaling, and chemotaxis [43]. More recently, it has been discovered that the PI3K/Akt pathway has a wide impact on the innate immune system [44]. Interestingly, the current work explained the effect of ischemic insult on apoptosis by the significant diminution of PI3K, Akt, and Ki-67 immunoexpressions, reflecting the occurrence of apoptosis with disturbances in cell proliferation and survival during JII/R. This is in harmony with Schmitz et al. (2020) and others who found that Ki-67 expression decreased after ischemic injury due to cell damage and apoptosis with suppression of cell renewal [45]. Also, previous research confirmed the great involvement of the PI3K/Akt/ki-67 pathway in ameliorating I/R-induced damage and the associated cardiac injury [46,47,48]. This hypothesis can be strengthened by the detected histological analysis, where several alterations were observed in the JII/R group, like derangement of myocardium fibers and band contraction, dispersed pyknotic nuclei, hyperemic blood vessels, and interstitial edema, as proved by other studies supporting our findings [49,50].

ET-1 is a vasoconstrictor agent that acts on ETA and ETB receptors in the vasculature and smooth muscle cells that express mainly the ETA subtype. Ischemia–reperfusion is associated with excessive release of ET and activation of its receptors. ERA expression was evaluated in the current model, and we found a significant increase in its expression in the JII/R model as previously detected in different studies. One of the selective ERA antagonists is AMB which has been approved in the treatment of pulmonary hypertension [51,52,53]. Despite the essential role of ET-1 in mediating myocardial damage and various ischemic injuries, there is no research discussing the potential ameliorative effect of AMB in JII/R. AMB could previously preserve the renal, pancreatic, cerebral, and hepatic vasculature during ischemia depending on its ability to control the ET receptor and decrease its vasoconstricting effect, antioxidant impact, anti-inflammatory role, and anti-apoptotic properties [19,20,21,23,54,55,56,57,58]. These pharmacological actions of AMB explain the already detected results, as it could decrease the levels of cardiac enzymes, cleaved Caspase-3, MDA, and NF-κB. In addition, there is a significant decrease in the highly expressed endothelin receptor A during JII/R, as this is the main pharmacological effect of AMB to antagonize ERA activity and expression. This is in accordance with different previous studies discussing the effect of AMB on endothelin receptor expression and activity [22,23,24]. However, there are significant increases in TAC and GSH levels, with an obvious improvement in the disturbed histopathological features and elevation of mTOR levels, PI3K, Akt, and Ki-67 immune expressions. AMB is primarily recognized as a selective ERA antagonist. However, receptor blockade is associated with subsequent modulation of the receptor expression. The ERA blockade of AMB is not the only action responsible for AMB’s effect, and different biological activities of ET-1/ERA signaling should be discussed to clarify the net effect of AMB. During ischemia/reperfusion injury, hypoxia occurs with excessive release of inflammatory mediators, including NF-κB, and the ET-1/ERA signaling mechanism becomes markedly activated, leading to enhanced inflammatory responses, oxidative stress, apoptosis, vasoconstriction, and tissue injury. This pathological activation also contributes to upregulation of ERA expression. There are two different endothelin receptors, ERA and ERB, that have opposing effects on the vasomotor tone, as ERA enhances long-acting vasoconstriction, while ERB promotes vasodilation. A growing body of literature suggests that ET-1 has a critical role in mediating endothelial and vascular smooth muscle dysfunction due to hypoxia/ischemia–reperfusion injury [22,23,24,49,50,51]. First, there is strong evidence that ET-1 promotes the activation of NADPH oxidase, which in turn stimulates the production of superoxide and other ROS in cardiomyocytes and vascular cells. Likewise, ROS themselves enhance ET-1 mRNA expression, therein creating a pathologic feedback loop that amplifies oxidative stress, which is a critical driver of hypoxia/ischemia–reperfusion injury via interference with NO-mediated vasodilation, promotion of apoptosis, and altered calcium homeostasis. In addition, ET-1 is an instrumental modulator of the vascular tone in microcirculations through its actions on vascular endothelial ERA and ERB receptors, triggering potent calcium and PKC-mediated vasoconstriction, and a variety of factors regulate ET-1 signaling, including PKC itself, HIF-1 alpha, NF-kB, TGF-beta, TNF-alpha, and microvascular shear stresses contributing to significant microvascular dysfunction. Furthermore, ET-1 enhances neutrophil adhesion to endothelial cells and promotes the production of chemotactic factors, particularly in ischemic injury. Therefore, blockade of ERA by AMB interrupts this pathological signaling cascade, resulting in attenuation of the inflammatory and oxidative pathways that are involved in sustaining ERA overexpression. Accordingly, the observed reduction in ERA following AMB treatment does not contradict the involvement of the ET-1/ERA axis; rather, it reflects secondary downregulation of receptor expression after suppression of ET-1-mediated signaling. Furthermore, AMB is a selective antagonist of ERA that does not inhibit ERB, thereby enhancing NO production, facilitating vasodilation, improving tissue oxygenation, and preventing tissue damage. These pleiotropic effects of AMB were previously discussed in different studies explaining our findings in the current model of the novel ameliorating role of AMB in the JII/R model [22,23,24,49,50,51].

The current research paves the first step towards considering AMB as an adjuvant medical treatment in JII/R. Further studies using AMB administration specifically before the reperfusion period, more relevant to the clinical situation, should be performed to emphasize the significance of AMB in treating JII/R. Given the conserved nature of the biological pathways investigated, the findings of this study are likely to be applicable to other mammalian species with comparable physiological and pathological responses, while direct extrapolation to humans should be made with caution.

## 4. Materials and Methods 

### 4.1. Animals

We used juvenile male rats of the Wistar albino species aged 3–4 weeks, weighing 80–100 g, from the National Center of experimental research, Giza, Egypt. All rats were regularly fed on a standard animal diet like chow and tap water and acclimatized in steel cages with wood-chip bedding. Each cage contained 3 rats exposed to a 12 h dark:light cycle, temperature of 24 ± 2 °C, and humidity of 30%. The Care and Use of Laboratory Animals Committee of the Faculty of Medicine, Aswan University, approved this study; No. Asw.Uni./1087/4/25. The study agrees with the universal EU Directive 2010/63/EU guidelines and ARRIVE guidelines.

### 4.2. Chemicals

AMB was obtained from EVA Pharm, Egypt. A total antioxidant capacity (TAC) kit was previously purchased from Biodiagnostic Co., Cairo, Egypt (Catalog # TA 2513). ELISA kits of troponin I, creatine kinase-MB (CK-MB), ERA, mTOR, lactate dehydrogenase (LDH), Akt, and PI3K (Catalog # MBS2033695, Catalog # MBS722833, CAT # MBS263783, Catalog # MBS7254602, Catalog # MBS2515061, Catalog # MBS1600201, and Catalog # MBS9518759, respectively) were obtained from My BioSource Co., San Diego, CA, USA, and the following antibodies were purchased for Western blotting evaluation: anti-NF-κB p65 antibody (L8F6, Cell Signaling Technology, Danvers, MA, USA, Catalog # 6956), anti-Caspase-3 antibody (Cell Signaling Technology, Danvers, MA, USA, Catalog # 9661), and anti-β-actin (Abcam, Cambridge, UK, Catalog # 8226). The ReadyPrep™ Protein Extraction Kit was purchased from Bio-Rad (Hercules, CA, USA, Catalog # 163-2086). The Bradford Protein Assay Kit was purchased from Bio Basic Inc. (Markham, ON, Canada, Catalog # SK3041). PI3K (Catalog # YPA2328), AKT (Catalog # A17909), and Ki-67 (Catalog # AI6919) antibodies were obtained from Biospes Chongqing, China, and ABclonal, Woburn, MA, USA, respectively.

### 4.3. Experimental Methodology and Design

All methods were performed in accordance with the universal EU Directive 2010/63/EU guidelines and ARRIVE guidelines. This was approved by the Care and Use of Laboratory Animals Committee of the Faculty of Medicine, Aswan University; No. Asw.Uni./1087/4/25.

According to pilot studies, previously published models, and the currently accepted pharmacokinetic profile of AMB, we gave AMB 3 h before ischemia (30 and 60 mg/kg), as maximum plasma concentration of AMB can be reached in 2–3 h, and the duration of ischemia was 30 min and reperfusion was 90 min. Moreover, this dose was chosen in relation to the recommended human dose [56,57,58]. 

Animal groups

The juvenile rats were separated into four equal experimental groups, with 10 rats in each group.

Group 1: The control (CON) group was given 1 mL of carboxymethyl cellulose 0.1% aqueous solution.

Group 2: The JII/R group received 1 mL of 0.1% carboxymethyl cellulose aqueous solution via an oral route, followed by laparotomy and superior mesenteric artery clamping for 30 min of ischemia and then 90 min of reperfusion.

Group 3: JII/R + AMB—low dose: AMB (30 mg/kg) [57] was given PO 3 h before II/R.

Group 4: JII/R + AMB—high dose: Rats received AMB (60 mg/kg) [57] PO 3 h before II/R.

AMB was adequately suspended in 0.1% carboxymethyl cellulose.

### 4.4. Surgical Steps

Isoflurane 5% was given for general anesthesia, then Betadine was applied for sterilization. A midline surgical incision was done, followed by clamping of the superior mesenteric artery for 30 min, then a reperfusion period for 90 min by removal of the clamps. The same steps were performed in the CON group without vessel occlusion [55].

### 4.5. Sample Collection

Humane endpoints were established to minimize animal suffering. Animals were monitored for clinical signs including weight loss, reduced mobility, abnormal posture, and loss of appetite. At the end of the study, each rat was physically euthanized by decapitation under anesthesia by isoflurane 5%. Blood was taken from the jugular neck veins and then centrifuged to obtain clear serum. We excised the small intestine and heart and then kept them in 10% formalin for histopathological examination, and a portion of the tissue was immediately frozen for biochemical measurement. Tissue was homogenized in 5 mL of phosphate-buffered solution (PBS) and then centrifuged at 5000 rpm for 15 min.

### 4.6. Detection of Cardiac Enzymes, ERA, PI3K, Akt, and mTOR by ELISA

Cardiac enzymes, ERA, and mTOR were assessed based on manufacturers’ instructions. The microtiter plate was pre-coated with its specific antibody, followed by adding suitable samples or standards. The proteins of interest were captured by their specific antibodies. Reactions were terminated by adding sulfuric acid, and color intensity was measured at 450–500 nm.

### 4.7. Measurement of Oxidative Stress Parameters

Detection of MDA, a specific parameter of oxidative stress, depends on the level of thiobarbituric acid, using a standard curve of 1,1,3,3-tetramethoxypropane [59]. Measurement of reduced glutathione (GSH), an antioxidant defensive agent, was based on the binding of the sulfhydryl group with Ellman’s reagent, forming a yellow color measured at 405 nm [60]. TAC evaluation is scientifically based on the reaction of the antioxidants in the obtained sample with a known amount of hydrogen peroxide, and the residual amount was evaluated at 510 nm.

### 4.8. Intestinal and Cardiac Tissue Histological Examination

Following decapitation and obtaining of blood samples, the heart and jejunum were immediately separated, fixed in 10% formalin solution, and processed into 5 µm thick paraffin sections, then stained with hematoxylin and eosin (H&E) [61]. The stained sections were examined blindly under the light microscope.

Staining technique for immunohistochemical studies: Staining was performed using polyclonal rabbit antibodies for PI3K, AKT and Ki-67. Paraffin sections of the heart and jejunum tissues in different groups were adequately cut into 5 μm thick sections and then incubated at 42 °C in the oven for 24 h. Then, these sections were deparaffinized in xylol for 1 h, hydrated in descending grades of alcohol, incubated in hydrogen peroxide for 5 min, and washed twice in PBS for 5 min each. The primary antibody in a dilution of 1:200 was added to the sections, which were incubated for about 1.5 h. Afterwards, these sections were washed twice in PBS for 5 min each. The secondary antibody in a dilution of 1–1000 was applied, and the tissue sections were further incubated for about 20 min; following that, they were washed three times in PBS for 5 min. Then, diaminobenzidine tetrahydrochloride solution was added to the sections with more incubation for 10 min. Then, the sections were adequately washed in distilled water, counterstained with Mayer’s hematoxylin for 2 min, washed in tap water, dehydrated, cleared, and mounted by DPX. Sections were evaluated blindly in relation to staining intensity.

Photography: An Olympus light microscope (Olympus, Japan) was used to evaluate the slides, and images of the histological and immunohistochemical sections were captured using an Olympus digital camera (U.TV0.5XC-3), and these images were saved as jpg.

#### 4.8.1. Morphometric Study

The heart tissue examination was scored according to the degree of damage as follows: 0 means no lesions; 1 is mild (1–25%); 2 is moderate (26–45%); and 3 is severe (>45%) [62]. Moreover, the assessment for mucosal damage in intestinal mucosa was scored [63] as follows: mucosal necrosis: no change (0), slight (1), moderate (2), or severe (3); mucosal destruction: no change (0), slight (1), moderate (2), or severe (3); mucosal infiltration: no change (0), slight (1), moderate (2), or severe (3); goblet cell loss: no change (0), slight (1), moderate (2), or severe (3).

#### 4.8.2. Measuring the Area Fraction of PI3K, Akt, and Ki-67

ImageJ 22 software (open-source Java image processing program) was used for detecting the area fraction of immune positivity in a standard measuring frame of 5 fields of view at ×400 magnification by a light microscope transferred to the monitored screen [64].

### 4.9. Western Blotting for Evaluating Caspase-3 and NF-κB p65 Protein Expression [65,66,67]

Total protein was extracted from homogenized juvenile intestinal and heart tissues using the ReadyPrep™ Protein Extraction Kit, following the manufacturer’s protocol. Protein concentrations in each sample were quantified via the Bradford assay using the Bradford Protein Assay Kit according to the manufacturer’s guidelines. For each sample, 20 μg of total protein was mixed with Laemmli sample buffer. An aliquot of 7.5 μg protein was denatured by boiling in Laemmli buffer for 5 min at 95 °C. Proteins were separated by sodium dodecyl sulfate–polyacrylamide gel electrophoresis and then transferred onto polyvinylidene difluoride membranes, and blocked for 1 h at room temperature in a blocking solution comprising 3% bovine serum albumin in tris-buffered saline with 0.1% Tween-20 (TBST). Then, membranes were incubated overnight at 4 °C with the appropriate primary antibodies (anti-NF-κB p65, anti-cleaved Caspase-3, and anti-β-actin) diluted in TBST. After primary antibody incubation, the membranes were washed three to five times for 5 min each with TBST and then subsequently incubated for 1 h at room temperature with horseradish peroxidase-conjugated secondary antibodies (goat anti-rabbit IgG-HRP, Novus Biologicals). Membranes were washed three to five times with TBST. Protein detection was performed using a chemiluminescence substrate (Clarity Western ECL Substrate, Bio-Rad, Hercules, CA, USA), and signals were detected by a CCD-based imaging system (ChemiDoc MP, Bio-Rad, USA).

Band intensities were quantified using Total Lab analysis software (Version 1.0.1, www.totallab.com). For each lane, the intensity of NF-κB p65 and Caspase-3 bands was normalized to the corresponding β-actin band from the same lane. Background subtraction and identical region-of-interest dimensions were applied consistently across all blots to ensure objective quantification. Preliminary calibration experiments were performed to determine the linear detection range of the chemiluminescence system. The protein load used for final experiments was 7.5 µg, selected because β-actin and all target proteins fell within the linear dynamic range under these exposure conditions. Pre-stained molecular weight markers were included on every gel to verify the expected sizes of NF-κB p65 (~65 kDa), Caspase-3 (~17–19 kDa for cleaved form), and β-actin (~42 kDa).

### 4.10. Statistical Analyses

The results were expressed as the mean +/− SEM that was determined by one-way analysis of variance using ANOVA and then Tukey’s multiple comparison test. The Shapiro–Wilk test was used to ensure normality and homogeneity. The detected *p* values less than 0.05 were significant, and GraphPad Prism software was used (version 5.01 for Windows, San Diego, CA, USA). The sample size of n = 10 rats in each experimental group was chosen according to the previous similar models, pilot studies, and the expected mortality rate to obtain a sufficient number of rats and power level 95%, effect size 0.8, confidence interval 95%, and *p* values less than 0.05 for significance using G Power 3.1 9.2 software (total number of rats was forty) [68,69]. A single animal was an experiment unit. All groups were compared to the control group and the JII/R group. All rats were male (n = 10 in each group); all animals were included in the study analysis, and no exclusion criteria were provided.

## 5. Conclusions

Ambrisentan administration ameliorated JII/R-induced injuries via modulation of ERA and increased the expression of PI3K, Akt, mTOR, and Ki-67 but decreased the expression of NF-κB and Caspase-3, reflecting its ability to improve cell proliferation and diminish inflammation, oxidative stress, and apoptosis.

### Study Limitations and Recommendations

The current study was limited by using a single species and sex, a relatively short follow-up, and a deficiency of dosing and long-term safety in the immature intestine. Furthermore, it is limited to assessing the plasma concentration of AMB, its specific pharmacokinetics in juvenile rats, and the functional protection of the intestine by detecting barrier function and bacterial translocation. We recommend performing more experimental and clinical studies to clarify these points in the further research plan.

## Figures and Tables

**Figure 1:**
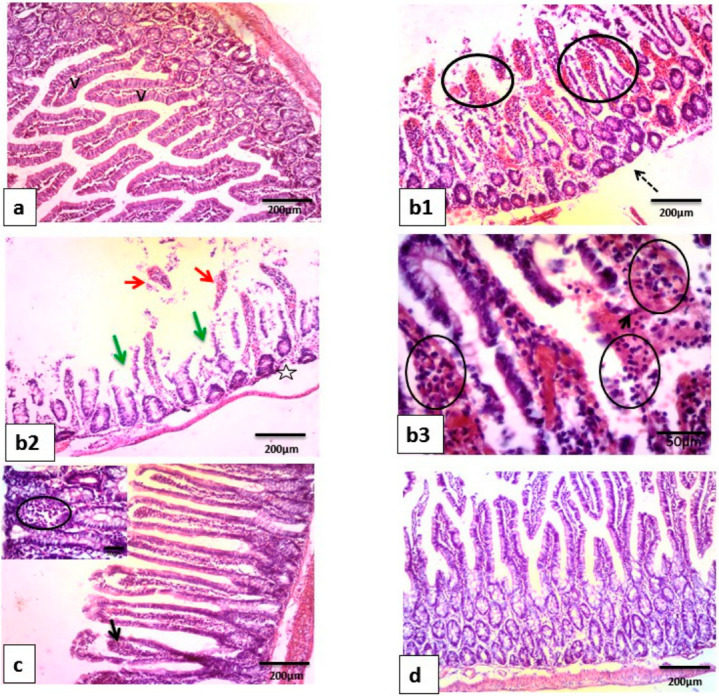
Histopathological examination of the intestinal tissue. Representative photomicrographs of the jejunum in the control (CON) group (**a**) showing normal intestinal mucosa with abundant finger-like villi (v). In the juvenile intestinal ischemia reperfusion (JII/R) group (**b1**–**b3**), severe mucosal tissue injury with loss of intestinal villi (red arrows), disintegration of the lamina propria (dotted arrow), hemorrhage with massive inflammatory cell infiltration (circles), mainly neutrophils (short arrow (**b3**)), apical denudation of the intestinal villi (green arrows), and epithelial separation with damage of the connective tissue (star) were observed. Treatment with a low dose of ambrisentan (AMB) improved most of these microscopic changes associated with JII/R, showing only some inflammatory cell infiltration (arrow and circle in inset (**c**)), while the high-dose AMB-treated group showed more or less normal jejunal histology (**d**). H&E 200× & 400× ((**b3**) and inset in (**c**).

**Figure 2:**
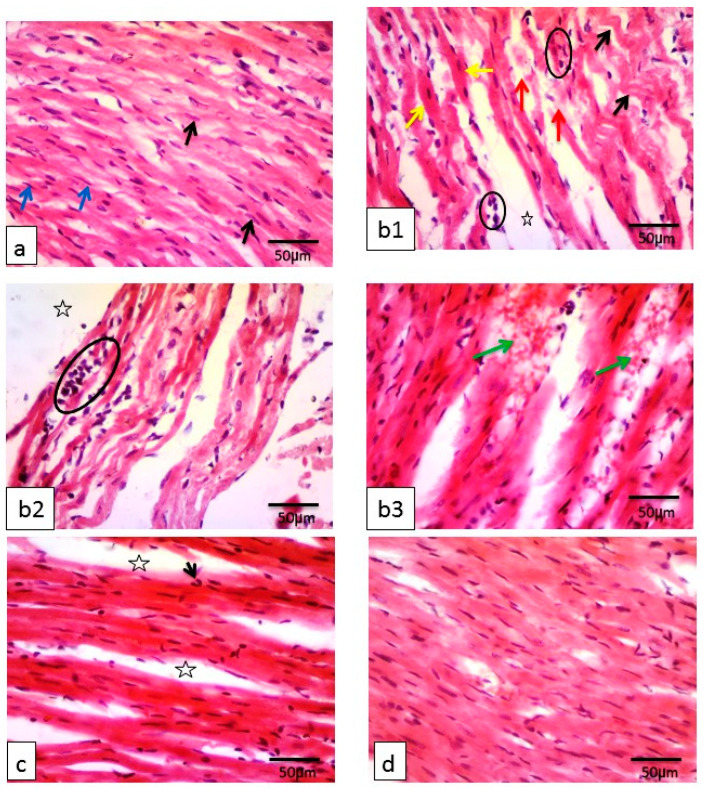
Histopathological examination of cardiac tissue. Photomicrographs of the left ventricle in the control (CON) group (**a**) showing normal individual cardiac muscle cells that were regularly arranged, anastomosed, and branched, forming a three-dimensional meshwork. Their sarcoplasm was acidophilic, striated, and had a single central oval vesicular nucleus (black arrows). Narrow spaces of connective tissue endomysium were seen between the cardiac muscle cells. Few fibroblasts with flat dense nuclei (blue arrows) were observed at the periphery of myocytes. In the juvenile intestinal ischemia reperfusion (JII/R) group (**b1**–**b3**), there were areas of degenerated muscle fibers (stars), inflammatory cell infiltration (circles), wavy fibers (black arrows), hypereosinophilic homogenous fibers with pyknotic deeply stained nuclei (yellow arrows), fragmented pale muscle fibers (red arrows), and vascular congestion (green arrows). The group treated with low-dose ambrisentan (AMB) showed improvement in most of these microscopic modifications associated with JII/R, showing less inflammatory cell infiltration ((**c**) arrow = neutrophil), wide areas of preserved cardiac fibers with central oval vesicular nuclei, and few areas of degenerated muscle fibers (stars). The high-dose-treated group (**d**) shows remarkably improved cardiac muscle histology ×400.

**Figure 3:**
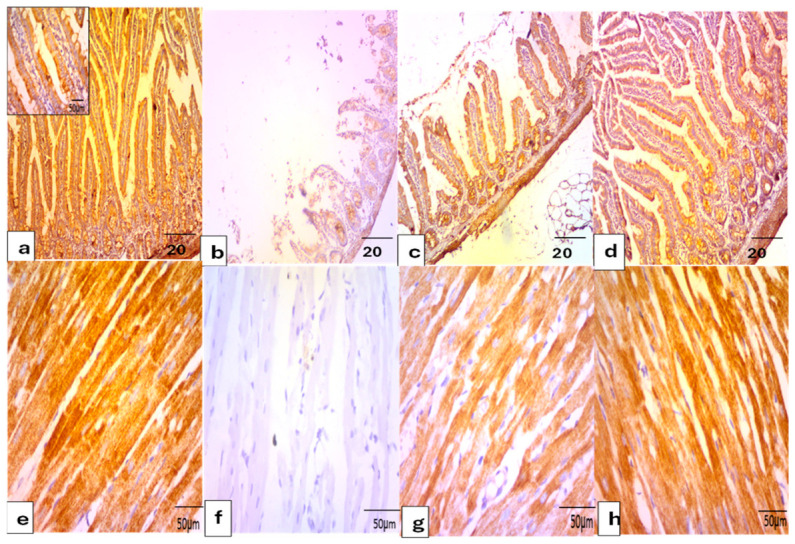
Results of phosphatidylinositol-3-kinase (PI3K) immunoexpression. Photomicrographs of the control (CON) group (**a**,**e**) immunohistochemically stained for PI3K showed positive cytoplasmic immune expression. The juvenile intestinal ischemia reperfusion (JII/R) group showed faint PI3K expression (**b**,**f**). The low-dose ambrisentan (AMB-LD)-treated group showed some restoration of positive immune expression in the cytoplasm (**c**,**g**). The high-dose ambrisentan (AMB-HD)--treated group showed strong PI3K immunoexpression (**d**,**h**). (IHC by PI3K antibody 200× and 400× (inset)).

**Figure 4:**
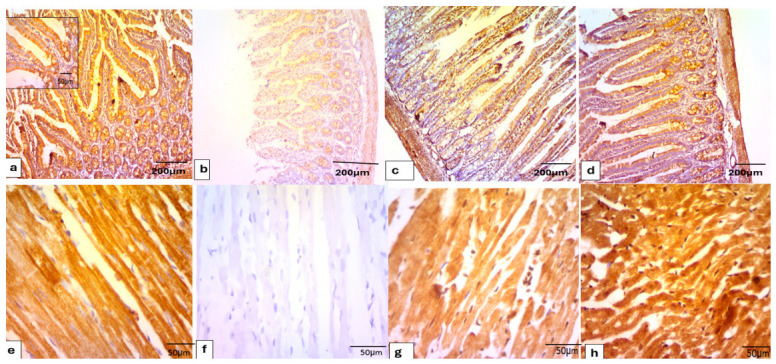
Results of protein kinase B (Akt) immunoexpression. Photomicrographs of the control (CON) group (**a**,**e**) immunohistochemically stained for Akt showed positive cytoplasmic immune expression but faint expression in the juvenile intestinal ischemia reperfusion (JII/R) group (**b**,**f**). The low-dose ambrisentan (AMB-LD)-treated group showed some restoration of positive immune expression in the cytoplasm (**c**,**g**), while the high-dose ambrisentan (AMB-HD) treated group showed strong expression (**d**,**h**). (IHC by AKT antibody 200× and 400× (inset)).

**Figure 5:**
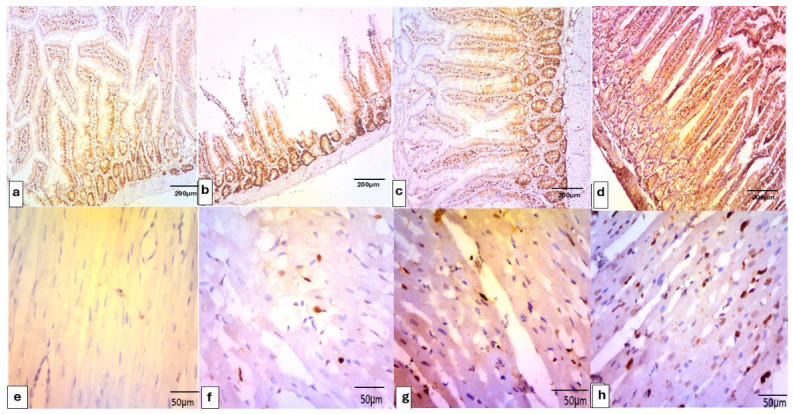
Results of Ki-67 immunoexpression. Photomicrographs of the control (CON) group (**a**,**e**) immunohistochemically stained for Ki-67 showed mild positive expression. On the other hand, faint Ki-67 expression was noticed in the juvenile intestinal ischemia reperfusion (JII/R) group (**b**,**f**). The low-dose ambrisentan (AMB-LD)-treated group showed more immune expression (**c**,**g**), while the high-dose ambrisentan (AMB-HD)-strengthened Ki-67 expression (**d**,**h**). (IHC by Ki-67 antibody 200× & 400× (insets)).

**Figure 6:**
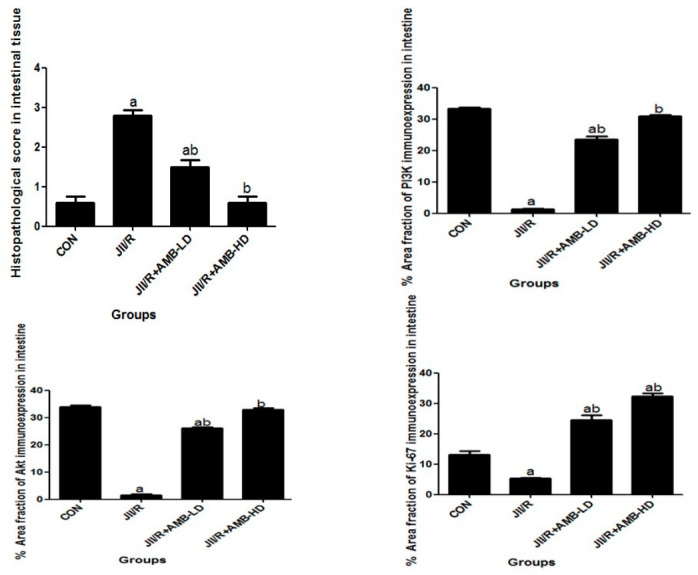
Semi-quantitative analysis of intestinal tissue immunohistopathological examination. CON is the control group, AMB-LD is ambrisentan low dose, AMB-HD is ambrisentan high dose and JII/R is juvenile intestinal ischemia–reperfusion group. The phosphatidylinositol-3-kinase (PI3K), protein kinase B (Akt), and Ki-67 immune expressions significantly decreased, but the histopathological score significantly increased in the JII/R group if compared to the CON group. Nevertheless, AMB-treated groups at low or high doses showed significantly increased PI3K, Akt, and Ki-67 expressions, while the histopathological score was decreased compared to the untreated JII/R group. Values of the study (n = 10) represent mean ± S.E.M. They are considered significantly different when the *p* value is less than 0.05. ^a^ Significant to CON group; ^b^ significant to JII/R group.

**Figure 7:**
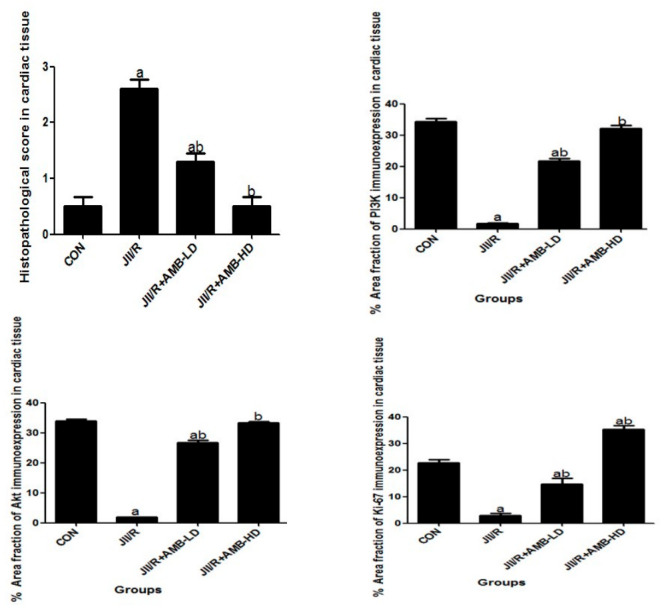
Semi-quantitative analysis of the cardiac tissue immunohistopathological examination. CON is the control group, AMB-LD is ambrisentan low dose, AMB-HD is ambrisentan high dose and JII/R is juvenile intestinal ischemia–reperfusion group. Phosphatidylinositol-3-kinase (PI3K), protein kinase B (Akt), and Ki-67 significantly decreased, but the histopathological score significantly increased in the JII/R group if compared to CON group. Nevertheless, AMB-treated groups at low or high doses significantly increased PI3K, Akt, and Ki-67 but decreased histopathological score compared to the JII/R untreated group. Values of the study (n = 10) represent mean ± S.E.M. that are significantly different when the *p* value is less than 0.05. ^a^ Significant to CON group; ^b^ significant to JII/R group.

**Figure 8:**
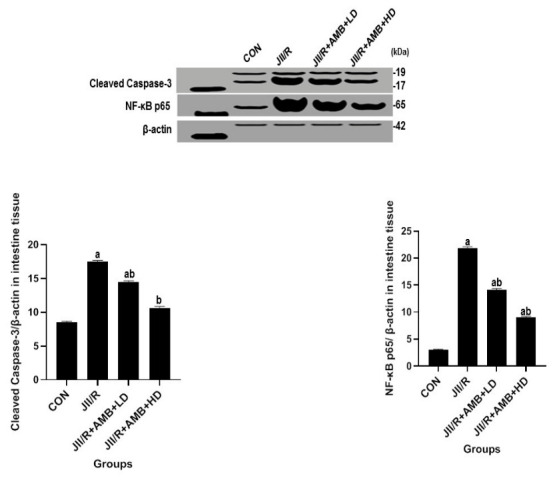
Western blotting expression of Caspase-3 and nuclear factor kappa b (NF-κB) p65 in intestinal tissue. CON is the control group, AMB-LD is ambrisentan low dose, AMB-HD is ambrisentan high dose and JII/R is juvenile intestinal ischemia–reperfusion group. Values of the study represent means ± S.E.M. that are significantly different when the *p* value is less than 0.05. ^a^ Significant to CON group; ^b^ significant to JII/R group. Phosphorylated NF-κB and cleaved Caspase-3 significantly increased in the untreated ischemic group of JII/R compared to the CON group. However, both parameters significantly decreased in the AMB-administered groups (30 and 60 mg/kg) if compared to the JII/R untreated group.

**Figure 9:**
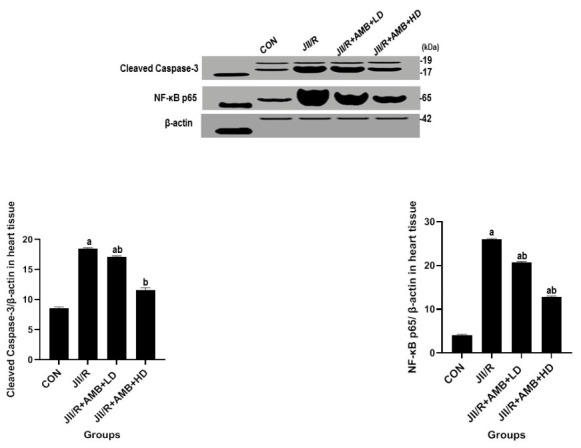
Western blotting expressions of Caspase-3 and nuclear factor kappa b (NF-κB) p65 in cardiac tissue. CON is the control group, AMB-LD is ambrisentan low dose, AMB-HD is ambrisentan high dose and JII/R is juvenile intestinal ischemia–reperfusion group. Values of the study represent means ± S.E.M. that are significantly different when the *p* value is less than 0.05. ^a^ Significant to CON group; ^b^ significant to JII/R group. Caspase-3 and NF-κB p65 significantly increased in the JII/R if compared to the CON group. However, they significantly decreased in the AMB-administered groups (30 and 60 mg/kg) relative to the JII/R untreated group.

**Table 1:** Impact of AMB on heart-specific enzymes in the JII/R model.

Groups	Troponin I (ng/mL)	CK-MB (U/L)	LDH (U/L)
**CON**	14.60 ± 0.8	16.5 ± 0.6	199.0 ± 3.2
**JII/R**	58.3 ± 2.5 ^a^	49.5 ± 1.9 ^a^	350.3 ± 10.4 ^a^
**JII/R + AMB-LD**	22.2 ± 1.0 ^ab^	38.1 ± 1.6 ^ab^	302.6 ± 11.5 ^ab^
**JII/R + AMB-HD**	17.42 ± 0.4 ^b^	20.5 ± 1.1 ^b^	252.0 ± 1.1 ^ab^

Values represent mean ± S.E.M. (n = 10) that are significantly different when the *p* value is less than 0.05. ^a^ Significant to CON group; ^b^ significant to JII/R group. CON is the control group, AMB-LD is ambrisentan low dose, AMB-HD is ambrisentan high dose, and JII/R is the juvenile intestinal ischemia–reperfusion group. CK- MB: Creatine kinase-MB, LDH: Lactate dehydrogenase.

**Table 2:** Oxidative stress parameters evaluation.

Groups	GSH in Intestine (µmol/g Tissue)	MDA in Intestine (nmol/g Tissue)	GSH in Heart (µmol/g Tissue)	MDA in Heart (nmol/g Tissue)	TAC in Serum (mmol/L)
**CON**	441.0 ± 16.8	29.3 ± 2.1	502.7 ± 10.2	33.5 ± 1.9	0.9 ± 0.02
**JII/R**	231.0 ± 10.6 ^a^	93.3 ± 5.6 ^a^	201.0 ± 15.9 ^a^	74.2 ± 2.4 ^a^	0.5 ± 0.03 ^a^
**JII/R + AMB-LD**	315.0 ± 25.8 ^ab^	69.8 ± 4.5 ^ab^	318.0 ± 18.9 ^ab^	47.4 ± 2.1 ^ab^	0.6 ± 0.03 ^ab^
**JII/R + AMB-HD**	473.0 ± 16.1 ^b^	39.1 ± 2.0 ^b^	475.0 ± 19.7 ^b^	34.0 ± 1.5 ^b^	0.7 ± 0.02 ^ab^

Values represent mean ± S.E.M. (n = 10) that are significantly different when the *p* value is less than 0.05. ^a^ Significant to CON group; ^b^ significant to JII/R group. CON is the control group, AMB-LD is ambrisentan low dose, AMB-HD is ambrisentan high dose, and JII/R is the juvenile intestinal ischemia–reperfusion group. MDA: Malondialdehyde, GSH: Reduced glutathione.

**Table 3:** Results of ERA and mTOR in tissues.

Groups	mTOR in Intestine (ng/mL)	ERA in Intestine (Pg/mL)	mTOR in Heart (ng/mL)	ERA in Heart (Pg/mL)
**CON**	2.1 ± 0.2	21.40 ± 1.6	1.9 ± 0.04	25.3 ± 1.4
**JII/R**	0.9 ± 0.06 ^a^	85.3 ± 3.4 ^a^	0.8 ± 0.03 ^a^	85.2 ± 3.1 ^a^
**JII/R + AMB-LD**	1.4 ± 0.1 ^ab^	74.7 ± 2.5 ^ab^	1.1 ± 0.05 ^ab^	66.5 ± 1.6 ^ab^
**JII/R + AMB-HD**	1.9 ± 0.02 ^b^	25.6 ± 1.2 ^b^	1.5 ± 0.07 ^ab^	33.8 ± 2.4 ^b^

Values represent mean ± S.E.M. (n = 10) that are significantly different when the *p* value is less than 0.05. ^a^ Significant to CON group; ^b^ significant to JII/R group. CON is the control group, AMB-LD is ambrisentan low dose, AMB-HD is ambrisentan high dose, and JII/R is the juvenile intestinal ischemia–reperfusion group. ERA: Endothelin receptor A, mTOR: Mammalian target of rapamycin.

**Table 4:** Results of PI3K and Akt in tissues.

Groups	PI3K in Intestine (ng/mL)	Akt in Intestine (ng/mL)	PI3K in Heart (ng/mL)	Akt in Heart (ng/mL)
**CON**	2.4 ± 0.02	2.1 ± 0.2	2.0 ± 0.1	1.4 ± 0.07
**JII/R**	1.1 ± 0.09 ^a^	0.9 ± 0.07 ^a^	0.9 ± 0.03 ^a^	0.7 ± 0.05 ^a^
**JII/R + AMB-LD**	1.6 ± 0.08 ^ab^	1.4 ± 0.09 ^b^	1.3 ± 0.02 ^ab^	0.9 ± 0.03 ^ab^
**JII/R + AMB-HD**	1.9 ± 0.01 ^ab^	1.5 ± 0.1 ^b^	1.8 ± 0.1 ^b^	1.5 ± 0.1 ^b^

Values represent mean ± S.E.M. (n = 10) that are significantly different when the *p* value is less than 0.05. ^a^ Significant to CON group; ^b^ significant to JII/R group. CON is the control group, AMB-LD is ambrisentan low dose, AMB-HD is ambrisentan high dose, and JII/R is the juvenile intestinal ischemia–reperfusion group. Akt: Protein kinase B, PI3K: Phosphatidylinositol-3-kinase.

## Data Availability

The datasets used and/or analyzed during the current study are available from the corresponding author on reasonable request.

## Abbreviations

AMB	Ambrisentan
Akt	Protein kinase B
CK-MB	Creatine kinase-MB
ERA	Endothelin receptor A
GSH	Reduced glutathione
JII/R	Juvenile intestinal ischemia–reperfusion
LDH	Lactate dehydrogenase
mTOR	Mammalian target of rapamycin
MDA	Malondialdehyde
NF-κB	Nuclear factor kappa b
PI3K	Phosphatidylinositol-3-kinase
ROS	Reactive oxygen species
TAC	Total antioxidant capacity
